# Supplementary material for: Transcriptomic Profiling Reveals AKR1C1 and AKR1C3 Mediate Cisplatin Resistance in Signet Ring Cell Gastric Carcinoma via Autophagic Cell Death
Source: Int J Mol Sci. 2021 Nov 19;22(22):12512. doi: 10.3390/ijms222212512 (PMC8623627; doi:10.3390/ijms222212512)
Supplement: Supplementary file 1 [file ijms-22-12512-s001.zip › ijms-1463097-supplementary.pdf]

Gene symbol	Forward Primer (5' –> 3')	Reverse Primer (5' –> 3')
AKR1C1	GCCATATTGATTCTGCTCATTTAT	TGGGAATTGCTCCAAAGC
AKR1C2	AAGTAAAGCTCTAGAGGCCGT	GCTCCTCATTATTGTAAACATGT
AKR1C3	TGGAAAACTCACTGAAAAAAGC	CATTTTCATCTGTTGGTGAAAG
AKR1B1	GGATGAAGGGCTGGTGAA	GTGGCACTCAATCTGGTTA
AKR1B10	CCCAAAGATGATAAAGGTAATGCCATCGGT	CGATCTGGAAGTGGCTGAAATTGGAGA
ALDOB	CACCATTCAAGGGCTTGATGGCCT	TTCCTGGATAGCGAGGCTGGAT
HSD12B2	CAATGCTGCAGGACAGAGGA	GTTCACGGCCATGCATTGTT
PLAC8	GGAACAAGCGTCGCAATGAG	AAAGTACGCATGGCTCTCCTT
FGF21	GACTGTGGGTTTCTGTGCTG	GAATAACTCCCGGCTTCAAGG
CPXM2	GTGCGCGGGAAGAAATGAC	CCTCCCTTGAGTGATGACACC
TBC1D4	AGATGGCCTGCCACGTT	CTCTTTCATGGCCGCTTTAG
FREM2	TGAGCCAACTGTGTTTATTC	GTATAACAGACCACCATCAAC
ALDH1L2	TAACACATACAACAAGACAGAT	ATATTCATTTAGAGCTTCCTCA
MDR1	CCCATCATTGCAATAGCAGG	GTTCAAACTTCTGCTCCTGA
MRP1	CGG AAA CCA TCC ACG ACC CTA ATC	ACC TCC TCA TTC GCA TCC ACC TGG
XRCC1	ACT GCT GGA ACC TGG CCC TGC	GCA AAC CCC GAG GAG AAG GCA
ERCC1	AAG TGC TGC GAG CCC TGG GC	AAT AAG GGC TTG GCC ACT CC
GAPDH	CCCCTTCATTGACCTCAACTAC	GATGACAAGCTTCCCGTTCTC

Table S1. Primer sequences used in this study were listed.

Top 50 upregulated genes	Log 2 Fold change	Top 50 upregulated genes	Log 2 Fold change
ANKS1B	10.00120701	*DPEP1*	4.010343936
TMEM200B	8.279903579	*NCF1*	3.976568327
TMOD1	7.411541132	*TNNI2*	3.975859619
CXCL13	6.582793746	*CTD-3193O13.9*	3.925505776
CLDN22	6.383369611	*LPPR1*	3.862463534
SLC10A2	6.221569778	*AKR1C3*	3.707357856
ALDOB	5.942942007	*HPGD*	3.704429688
HSD17B2	5.63596137	*SRGAP3*	3.667642121
PLAC8	5.5946095	*GGTLC2*	3.654972786
CTD-3074O7.11	5.579142468	*CYP4F12*	3.639574743
AKR1C1	5.565227132	*PDZD3*	3.553896042
APOBEC1	5.543903602	*KRT20*	3.484469377
ENPP3	5.381785846	*GAGE12F*	3.474989902
AKR1C2	5.295546827	*GAGE12G*	3.471310628
ADAMTS18	4.813060175	*PADI2*	3.470230902
RP11-6N17.6	4.74912944	*CRYBB1*	3.468425963
DEFB1	4.716958372	*LGALS9C*	3.395554164
LGALS2	4.546300171	*APOD*	3.331868841
PHGR1	4.412965345	*ACSM3*	3.295612591
FAM3D	4.393154683	*CDH17*	3.280257658
REG4	4.376451913	*HTN1*	3.254720797
TM4SF5	4.338300867	*AKR1B15*	3.247006572
SPINK4	4.20820479	*LINC01088*	3.245920558
UGT1A5	4.190219093	*NMUR2*	3.240752369
PART1	4.146625076	*TGFBR3L*	3.187284262

Table S2. Top 50 upregulated differentially expressed genes (KATO/DDP vs KATOIII) analyzed by CLC genomic work bench.

Top 50 down regulated genes	Log 2 Fold change	Top 50 down regulated genes	Log 2 Fold change
DUX4L7	-10.08529421	*TBC1D4*	-5.471998485
MSANTD4	-9.938113925	*ANKRD20A3*	-5.420006916
GCOM1	-9.004911443	*SLITRK1*	-5.303238935
ZNF134	-8.908232759	*ASB3*	-5.17177588
TUBA3C	-8.880048623	*LUC7L2*	-5.048251183
GAGE12C	-8.751851805	*ALDH1L2*	-4.990809922
TUBA3E	-8.72756069	*CT45A5*	-4.978625427
RP11-544M22.13	-7.961757733	*PRSS1*	-4.975307056
PCYT1B	-7.959000431	*FAM157B*	-4.965680434
ATP10A	-7.847628147	*NECAB2*	-4.931482747
GAGE12D	-7.638452541	*GAGE2C*	-4.918040046
OLFM2	-7.340402539	*TGFB2*	-4.917914633
TNFRSF9	-7.267109125	*FREM2*	-4.913189506
DUX4L13	-6.65803919	*EYA4*	-4.90850198
FAM115B	-6.510186858	*ZNF469*	-4.884203884
PRSS3P2	-6.415960084	*SSX1*	-4.829520825
LINC00326	-6.096395261	*TBX20*	-4.829215923
FGF21	-6.052656406	*SOHLH2*	-4.744286679
ST6GALNAC5	-6.031810226	*SOX5*	-4.539827753
GDAP1L1	-5.829606389	*PLAC9*	-4.517630046
GGN	-5.823501787	*FAM200A*	-4.465701387
CPXM2	-5.707164065	*HCK*	-4.456694213
DUX4L4	-5.686992478	*THSD7A*	-4.455646016
POTEF	-5.666120276	*POTED*	-4.437075994
GAGE12J	-5.518564693	*PDE4B*	-4.436759955

Table S3. Top 50 downregulated differentially expressed genes (KATO/DDP vs KATOIII) analyzed by CLC genomic work bench.

Gene ontology biological process term	Genes Names
**Oxidation reduction process**	***STEAP2, ALDH1L2, AKR1B10, AKR1B15, AKR1C1, AKR1C2, AKR1C3, CBR3, CYP1A1, Cyp26B1, CYP4F12, CYP4F3, FAR2P1, HPGD, HSD17B2, IDP1, LPO, LOXL4, NCF1, PDPR, SOD3***
**Digestion**	***UGT1A1, ADM2, AKR1B10, AKR1C1, AKR1C2, PRSS1, PRSS3P2, TFF2, TFF3***
**Positive regulation of gene expression**	***PRDM1, SMAD1, WNT11, ADM2, ANK2, CAV1, CYP26B1, HMGN5, RPS6KA2, SLC26A9, TGFB2, VEGFA***
**Angiogenesis**	***ADM2, APOD, CAV1, NRXN1, RAMP1, SLC12A6, THSD7A, TGFB2, TNFSF12, UNC5B, VEGFA***
**Negative regulation of cell proliferation**	***CD33, ETS1, FOSL1, KLF10, SMAD1, TNFRSF9, CDKN2B, IFITM1, RARRES3, RPS6KA2, SSTR5, TES, TGFB2***
**Steroid metabolic process**	***UGT1A1, AKR1B10, AKR1C2, AKR1C3, CYP1A1, SULT1E1***
**Regulation of apoptosis process**	***BCL2L14, ETS1, IKZF3, TNFRSF9, CASP1, CARD16, DAPK1, INHPC, INHBE, SDF2L1***
**Daunorubicin metabolic process**	***AKR1B10, AKR1C1, AKR1C2, AKR1C3***
**Doxorubicin metabolic process**	***AKR1B10, AKR1C1, AKR1C2, AKR1C3***
**Response to hypoxia**	***DTIT4, ETS1, CA9, CASP1, CAV1, CYP1A1, SOD3, TGFB2, VEGFA***

Table S4. Genes involved in top ten gene ontology biological process by David gene function annotation.

GO term	Upregulated genes (KATO/DDP vs KATOIII)	Downregulated genes (KATO/DDP vs KATOIII)
**Oxidation reduction Process**	***ALDOB, AKR1C1, HSD17B2, AKR1C2, NCF1, CYP4F12, AKR1C3, HPGD, APOD, AKR1B15, CYP4F3, IDO1, AKR1B10, CBR3***	***SESN2, SOD3, MT-ND4L, LOXL4, STEAP2, PDPR, CYP1A1, CYP26B1, LPO, ALDH1L2***
**Small molecule metabolic process**	***ALDOB, AKR1C1, APOBEC1, ENPP3, AKR1C2, UGT1A5, DPEP1, CYP4F12, AKR1C3, HPGD, ADOP, ACSM3, TCN1, CYP4F3, IDO1, AKR1B10, CBR3, QPRT, UGT1A1, TFF3, UGT1A10, BAAT, PSMB10, DBI***	***SESN2, ABCA1, PDPR, CAV1, CYP1A1, GSTM2, OGT, CYP26B1, CTH, RIMKLB, SULT1E1, ST6GAL1, LPO, PDE4B, PRSS1, ALDH1L2***
**Cofactor metabolic process**	***ALDOB, AKR1C1, AKR1C2, DPEP1, GGTLC2, AKR1C3, ACSM3, TCN1, IDO1, AKR1B10, CBR3, QPRT, UGT1A1, BAAT, DBI***	***PDPR, CYP1A1, GSTM2, CHAC1, LPO, PRSS1, ALDH1L2***

Table S5. Genes involved in top GO terms by gene set enrichment analysis
